# Gender differences regarding problematic smartphone use, mediating by mental well-being, emotional regulation, and social anxiety in young adult sample

**DOI:** 10.1192/j.eurpsy.2025.2430

**Published:** 2025-08-26

**Authors:** C. Sándor, N. Pirwani, M. Csibi, A. Szabo

**Affiliations:** 1 Faculty of Psychology, Department of Sciences and Letters, George Emil Palade University of Medicine, Pharmacy, Science, and Technology, Targu Mures, Romania; 2 Institute of Health Promotion and Sport Sciences, Faculty of Education and Psychology, ELTE Eötvös Loránd University, Budapest; 3 Institute of Special Education, Faculty of Pedagogy, Eszterházy Károly Catholic University, Eger, Hungary

## Abstract

**Introduction:**

Research underlay various results concerning the differences focusing on gender in smartphone use and mental issues. Some find no difference, but assessing mental factors reveals the influence of social interactivity, low emotional understanding, and perceived social support variations according to problematic smartphone use.

**Objectives:**

Our research aims to investigate problematic smartphone use, mental well-being, emotional regulation, and social anxiety as a function of gender to explore specific ways of functioning in the addiction process.

**Methods:**

The study participants were 400 young adults, of whom 104 were men (26%), 293 were women (73.2%), and three individuals (0.8%) were of another gender. The mean age of the participants was 25.9 years (SD 10.9). Registered answers refer to demographic data (gender, age, smartphone usage habits) as well as psychological measures: a Smartphone Application-Based Addiction Scale (SABAS), a Mental Health Continuum Scale (MHC), an Assessing Emotions Scale (AES), and Fear of Negative Perception Questionnaire (FNPQ).

**Results:**

The results showed that gender showed significant differences in the mediating factors that affect problematic smartphone use. In the mediation model, gender relates significantly related to time spent with smartphones (p=.001) and fear of negative perception (p=.001). The statistical mediation model highlighted the gender-depending significant role of the mediating factors (time of use per day, MHC, AES, FNPQ) in problematic smartphone use.

**Conclusions:**

Our research highlights gender differences in excessive smartphone use, such as higher social anxiety, which is a higher predisposing factor in women than in men. Gender is a significant indirect determinant of problematic smartphone use.

**Disclosure of Interest:**

None Declared

